# Integration of Digital Twin Technology in Orthodontics: A Scoping Review

**DOI:** 10.1155/ijod/3347380

**Published:** 2026-06-20

**Authors:** Isha Bhardwaj, Nidhin Philip Jose, Supriya Nambiar, Dhruv Ahuja, Shushma Rao, Sabarinath Prasad

**Affiliations:** ^1^ Department of Orthodontics and Dentofacial Orthopaedics, Manipal College of Dental Sciences Mangalore, Manipal Academy of Higher Education, Manipal, 576104, Karnataka, India, manipal.edu; ^2^ Department of Orthodontics and Dentofacial Orthopaedics, Manav Rachna Dental College, Manav Rachna International Institute of Research and Studies (MRIIRS), Faridabad, Haryana, India, mrdc.ac.in; ^3^ Department of Public Health Dentistry, Manipal College of Dental Sciences Mangalore, Manipal Academy of Higher Education, Manipal, 576104, Karnataka, India, manipal.edu; ^4^ Department of Orthodontics and Dentofacial Orthopaedics, Mohammed Bin Rashid University of Medicine and Health Sciences (MBRU), Dubai, UAE, mbruniversity.ac.ae

**Keywords:** artificial intelligence, malocclusion, orthodontist, personalized medicine

## Abstract

**Background:**

Digital twin technology (DTT) is an emerging concept in healthcare enabling real‐time patient and process simulations and predictive analytics that enhance personalized treatment and clinical decision‐making. Orthodontics, being a comprehensive subject involving technicalities associated with diagnosis and treatment planning, the specific role of DTT, remains underexplored. This gap presents a crucial opportunity to harness its power to create dynamic virtual models for truly personalized and data‐driven care.

**Objective:**

The objective is to map the extent and type of evidence available on the applicability of DTT in orthodontics, including its advantages, challenges, implementation in clinical settings, and perspective trends.

**Methods:**

A review synthesis for relevant articles published between 2005 and April 2025 in the English language was conducted across electronic databases including PubMed, Scopus, Embase, and Web of Science. Studies meeting the predefined eligibility criteria were included, and data relating to the applicability of digital twins (DTs) in orthodontics were assessed. To identify trends and inconsistencies in the existing literature, data extraction and synthesis of the findings were performed following the Joanna Briggs Institute (JBI) methodology.

**Results:**

This scoping review has collated a consolidated view of research investigating the applicability of the DTs in orthodontics specifically. Nine studies were identified that mapped applications of this technology in orthodontics, which are currently limited yet diverse, ranging from static craniofacial twins built from cone beam computed tomography (CBCT) to multimodal ones combining CBCT, intraoral scanner (IOS), and facial scans for more detailed analysis. The elaborate theoretical foundation available for this technology in other sectors has the potential to be executed in orthodontics to revolutionize the field.

**Conclusions:**

DTT exhibits immense potential and clinical significance in the field of orthodontics and dentofacial orthopedics in the nearing future to ultimately enhance the accuracy and efficiency of diagnosis and treatment like never before, improving overall experience for the clinicians, students, and the patients.

## 1. Introduction

Digital twin technology (DTT) is an emerging concept that integrates real‐time data with virtual simulations to optimize treatment planning, monitoring, and decision‐making processes across a variety of fields, including engineering and healthcare [1]. Despite digital twins (DTs) having the promising potential to revolutionize patient care in orthodontics, the exact applications remain underexplored. The creation of constantly updated, patient‐specific digital models that predict outcomes and improve care holds great promise in the advancement of fields like healthcare. While traditional digital tools like cone beam computed tomography (CBCT), intraoral scanners (IOSs), three‐dimensional (3D) printing, and artificial intelligence (AI) [2] have already enhanced diagnosis and treatment [3] and laid the groundwork for DT application [4–7], the DTs take it a step further by continuously integrating multiple sources of patient data. Although explicit research on DTs in orthodontics is still limited, studies in related areas such as healthcare [8, 9], maxillofacial surgery [10], radiology [11], and AI‐driven treatment models show the potential for adopting this technology to provide more personalized and precise orthodontic care [12]. Additionally, advancements in CBCT imaging, intraoral scanning, and computational fluid dynamics have contributed to more accurate patient‐specific simulations, laying the groundwork for DT applications [4–7].

A DT is a virtual representation of a physical entity or system that leverages real‐time data to precisely mirror the actual object’s behavior, performance, and state. Its three key elements are data acquisition, data modeling, and data application. The concept primarily harnesses four technologies to gather and store real‐time data, extract valuable insights, and create a digital replica of a physical asset—Internet of Things (IoT) connects devices and sensors to collect real‐time data from physical objects. AI analyzes and interprets the real‐time data, offering predictions, insights, and intelligent decision support. Extended reality (XR) merges virtual and physical environments using augmented reality (AR), virtual reality (VR), and mixed reality (MR), thereby allowing interactive visualization of digital replicas [1, 7, 12]. Cloud computing stores and processes massive data sets efficiently, enabling fast access and scalable computation—providing a continuous connection between the physical and cyber systems, enabling seamless synchronization and interaction. The extent to which these technologies are used varies depending on the specific application.

Until now, several studies have explored DTT in dentistry, revealing growing interest in its applications, and although these remain relatively limited, its focus in orthodontics is even more sparse. For instance, Cho et al. [5] emphasized that DTs enable a highly personalized approach by integrating CBCT and facial scan data for diagnosis and treatment planning. Pesapane et al. [11]) highlighted the use of AI‐powered DTs in radiology that enhance diagnostic accuracy and equipment maintenance. Even hybrid models combining CBCT and intraoral scans provide effective DTs usable as alternatives to traditional orthodontic models, although their developmental level is not as high on the hierarchy as many others [7]. By lowering the interexaminer variability and enabling patient‐specific customizations, the digital models including intraoral scans and CBCTs greatly improve diagnostic precision and treatment planning [13, 14]. A study by Ma et al. [12] described the potential applications of DTT in orthodontics. However, despite this promising evidence, there remains a scarcity of comprehensive studies providing a conclusive path for the integration of DTs into orthodontic procedures—highlighting the need for a dedicated scoping review to map all existing knowledge and guide future research in this emerging area.

By mapping the available evidence, our synthesis aims to identify research gaps, clarify key concepts, and explore the feasibility and challenges of integrating DTs into orthodontic practice. The objective is to assess the extent and nature of existing literature on DTT in orthodontics, emphasizing on the perspective trends emerging—including its application in personalized treatment planning, predictive modeling, and precision medicine. Given the interdisciplinary nature of this revolutionary technology, there is a need to consolidate current knowledge and understand its applicability in orthodontics. This review has synthesized these findings to assess the feasibility and current state of DT adoption in orthodontics and will serve as a foundation for future empirical research and clinical applications in the field.

## 2. Methodology

### 2.1. Study Design

This scoping review was conducted in accordance with the PRISMA ScR Checklist (Supporting Information Table S1) for scoping reviews [15–17].

Studies were examined using the PCC framework.

Participants: Studies that include DTT in orthodontic planning and procedures, orthodontists, and patients of all ages undergoing orthodontic treatment will be included in this study, while studies on DTs relating to other dental specialties, health professions, and nonhealthcare industries will be excluded from this study.
Concept: The concept of interest is the DTT in orthodontics. This includes virtual patient modeling, AI‐assisted treatment simulations, and predictive analytics that would enhance the orthodontic workflow.
Context: The context is the field of orthodontics and dentofacial orthopedics.

The formulated research question was as follows: What does the existing evidence reveal about the applicability of DTT in orthodontics and dentofacial orthopedics?

The review protocol is registered on OSF: https://doi.org/10.17605/OSF.IO/3QFKJ.

### 2.2. Search Strategy

Electronic databases including PubMed, Scopus, Embase, and Web of Science were searched to screen published articles. Google Scholar was also used to search for gray literature. The search strategy included the following MeSH terms: (“orthodontists”[MeSH Terms] OR “Orthodontist”[Text Word] OR (“dentists”[MeSH Terms] OR “Dentist”[Text Word]) OR (“malocclusion”[MeSH Terms] OR “malocclusion”[Text Word])) AND (“digital twin”[Text Word] OR “digital model”[Text Word] OR “virtual twin”[Text Word] OR “twin model”[Text Word] OR “twin technology”[Text Word] OR “digital twin technology”[Text Word] OR (“precision medicine”[MeSH Terms] OR “personali ^∗^ed medicine”[Text Word]) OR “virtual replica”[Text Word]) AND (“Orthodontics”[MeSH Terms] OR “Orthodontics”[Text Word] OR “dentofacial orthopaedics”[Text Word] OR (“Orthognathic Surgery”[MeSH Terms] OR “orthognathic”[Text Word]) OR (“dentistry”[MeSH Terms] OR “dentistry”[Text Word])) (Supporting Information Table S2).

### 2.3. Eligibility Criteria

Findings only applied to studies conducted in the last 20 years, that is, from 2005 to the present year, 2025, in the English language. The reason for selecting this time range is the need for assessing the roots of this technology as it was first conceptualized and developed in the early 2000s, primarily in engineering and manufacturing industries, before making its way into dentistry, including orthodontics, where it has been rather underexplored so far. The criteria were carefully designed to focus on studies directly related to the applicability of DTT in the field of orthodontics following the PCC framework. The integration of this technology in other sectors such as general healthcare, manufacturing, aerospace, and construction was excluded. This review considers descriptive observational study designs including case series, individual case reports, and review studies for inclusion.

### 2.4. Data Extraction and Quality Assessment

To develop a comprehensive search strategy, a preliminary search of PubMed was performed to scrutinize the text words found in the titles and abstracts, including any index keywords. Second, a second search across PubMed, Scopus, Embase, and Web of Science was carried out using all specified keywords and index terms. Third, the reference lists of all accessed articles were checked for additional studies and were included, as considered eligible. Furthermore, ProQuest Dissertations and Theses and Google Scholar were used to search for studies that would not have been easily accessed through conventional databases. A thorough two‐reviewer approach was employed to ensure the inclusion of relevant studies. Abstracts that did not provide enough information for inclusion were excluded. Additionally, the reference lists of the selected articles were manually reviewed to identify any potentially relevant studies missed during the initial database search. After selection, two researchers (I.B. and N.P.J.) independently extracted data from the included studies.

Following the search, all identified citations were collated and uploaded into Rayyan software [18] (http://rayyan.qcri.org), and duplicates were removed. Two independent reviewers (I.B. and D.A.) collaborated to identify and evaluate articles from different databases. They screened the titles and abstracts of each citation. Potentially relevant sources were retrieved in full, and their citation details were imported into the Joanna Briggs Institute (JBI) System for the Unified Management, Assessment and Review of Information (JBI SUMARI) (JBI, Adelaide, Australia) [19]. The full text of selected citations was assessed in detail against the inclusion criteria by the two independent reviewers. During full‐text screening, some articles proved to be focusing more on DTs’ involvement in the medical field and other dental branches without description of any aspects that would be relevant in orthodontic processes and hence were subsequently excluded. Any disagreements that arose between the reviewers at each stage of the selection process were resolved with discussion. The three‐stage search process proposed by JBI was used to determine the eligibility of published studies [15].

### 2.5. Data Analysis and Presentation

The evidence is presented in tabular form in Table 1. Coding and thematic analyses were conducted using ATLAS.ti software version 23 for Mac (ATLAS.ti Scientific Software Development GmbH: ATLAS.ti Mac (Version 23.2.2.27458) [Computer program] 2023). A narrative summary accompanies the tabulated and charted results and describes how the results relate to the review’s objective and the research question (Table 1).

**Table 1:** Thematic analysis of the articles.

S. number	Themes	Subthemes	Codes	References
1	**Basic conceptual foundation of DTT**	**Historical perspective and context**	DT in aerospace and shift to healthcare	De Domenico et al. [20] and Grieves [21]
			Early simulation models	Cho et al. [4]
			Evolution To dynamic systems	Liu et al. [22]
			Emergence In orthodontics	Ma et al. [12]
			COVID‐19 pandemic as a drive for accelerated development	Attaran and Celik [1] and Moztarzadeh et al. [23]
		**Definitions**	DT as an active unique product (Encyclopaedia of Product Engineering, 2019)	Attaran and Celik [1]
			DT as a virtual replica (Michael Grieves, 2003)	Grieves [21]
			DT as real‐time digital representation (Fu et al, 2022)	Attaran and Celik [1]
			DT based on data integration (Kritzinger et al, 2018)	Attaran and Celik [1]
			DT as a set of virtual models [24]	Liu et al. [22]
			DT as behavioral biological model	Semeraro et al. [25]
		**Status in the market**	Top 10 healthcare technology trends	Ma et al. [12]
			Gartner survey findings (2019, 2022, and 2023)	Attaran and Celik [1]
			Global Market Insight analysis (2022)	Attaran and Celik et al. [1]
			Adoption trend in the industry	Pesapane et al. [11] and Cho et al. [5]
			Private sector investment surge	Moztarzadeh et al. [23]
			Government‐led pilot programs	Liu et al. [22]

2	**Technologies used by DTT**	**Data acquisition technologies**	3D scanning	Cho et al. [4] and Liu et al. [22]
			CBCT imaging	Ma et al. [12], Cho et al. [4], and Lee et al. [7]
			Photogrammetry	Ma et al. [12] and Lee et al. [7]
			MRI integration	Pesapane et al. [11] and De Domenico et al. [20]
		**Modeling and simulation tools**	Finite element analysis	Semeraro et al. [25]
			Multibody dynamics	Cho et al. [4]
			Artificial intelligence–based segmentation	Liu et al. [22] and Cho et al. [5]
			Simulation engines	Liu et al. [26]
			Integration with virtual reality, mixed reality, and augmented reality	Ma et al. [12]
		**Data management and communication**	Internet of Things	Attaran and Celik [1], De Domenico et al. [20], and Ma et al. [12]
			Cloud computing	Grieves et al. [21] and Ma et al. [12]
3.	**Application prospects in orthodontics**	**Diagnosis and treatment planning**	Personalized medicine	Liu et al. [22] and Ma et al. [12]
			Growth prediction	Lee et al. [7] and Liu et al. [22]
			Skeletal maturity indicators, CVMI, and timing	Ma et al. [12]
			Cephalometric analyses	Ma et al. [12]
			Treatment approach testing	Semeraro et al. [25] and Cho et al. [5]
		**Appliance design and optimization**	Aligner fit simulation	Cho et al. [4]
			Bracket positioning	Lee et al. [7] and Liu et al. [22]
			Force calibration	Semeraro et al. [25]
		**Treatment simulation**	Orthodontic treatment planning	Cho et al. [4] and Semeraro et al. [25]
			Predictive modeling	Liu et al. [22] and Ma et al. [12]
			Craniofacial growth forecasting	Cho et al. [4] and Liu et al. [22]
		**Monitoring and feedback systems**	Remote progress tracking	Ma et al. [12]
			Behavioral tracking	De Domenico et al. [20] and Attaran and Celik [1]
		**Education and training**	Simulation for trainees	Attaran and Celik [1] and Cho et al. [5]
			Error‐free repetition	Attaran and Celik [1]
			Anatomical variability exposure	Cho et al. [5]
			Noninvasive learning	Attaran and Celik [1]
			Curriculum integration	Attaran and Celik [1]

4.	**Limitations and challenges**	**Technical barriers**	Data accuracy limitations	Cho et al. [5] and Liu et al. [26]
			Processing requirements and complexity of data integration	Grieves [21] and De Domenico et al. [20]
			Lack of standardization	Ma et al. [12], Semeraro et al. [25]
			High computing demand	Moztarzadeh et al. [23] and Liu et al. [22]
			Model fidelity concerns	Liu et al. [22] and Cho et al. [5]
			Concerns regarding standardization	Moztarzadeh et al. [23] and Attaran and Celik [1]
		**Ethical and regulatory concerns**	Data privacy risk	Attaran and Celik [1] and Pesapane et al. [11]
			Informed consent	Grieves [21]
			Accountability ambiguity	Liu et al. [22]
			Cross‐border regulations	Moztarzadeh et al. [23]
		**Clinical integration challenges**	Training gap	Ma et al. [12] and Liu et al. [22]
			Cost implementation	De Domenico et al. [20] and Iwasaki et al. [6]

5.	**Future directions**	**Interdisciplinary integration**	DTT AI synthesis	Liu et al. [22] and Cho et al. [4]
			Neuroscience integration	Semeraro et al. [25]
		**Clinical trial validation**	Need for randomized control trials	Ma et al. [12] and Attaran and Celik [1]
			Measures of outcomes	Grieves [21]
		**Personalized orthodontics**	Adaptive simulation loops	Semeraro et al. [25] and Cho et al. [5]
			Genomics integration	Cho et al. [4]
			Microbiome simulation	Liu et al. [22]
			Behavioral feedback loops	Liu et al. [22]
			Patient‐specific algorithms	Cho et al. [5]
			Universal interoperability	Grieves [21] and Moztarzadeh et al. [23]
		**Infrastructure and standards**	Blockchain security models	Pesapane et al. [11]
			Open source frameworks	Attaran and Celik [1]
			Real‐time analytics integration	Liu et al. [22]

*Note:* The bold values are the themes and subthemes under which information was broadly found to be described in the articles studied.

## 3. Results

**Figure 1:**
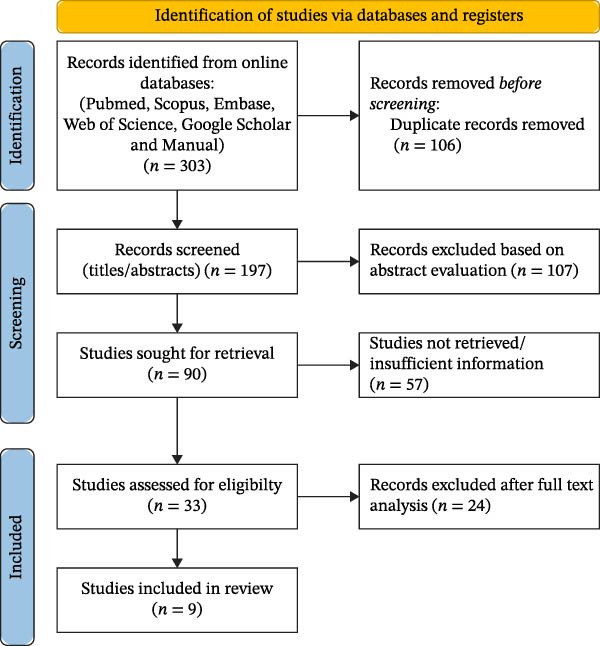
PRISMA ScR flow diagram.

The selection process utilizes a PRISMA flow diagram to illustrate the article selection process. After eliminating duplicates and irrelevant abstracts across the databases, 197 studies underwent initial screening by two independent reviewers. Any discrepancies in inclusion were resolved through discussion to reach a consensus. Ultimately, nine scientific articles aligned with the review’s objectives and were chosen for further analysis (Figure 1).

The electronic search, guided by the PCC framework, identified nine relevant studies. This scoping review adopted a rigorous approach, avoiding simplifications in data analysis. We focused on the research specifically employing DTT in orthodontic investigations. Table 2 summarizes the key findings from the included studies.

**Table 2:** Literature review on applicability of digital twin technology in orthodontics.

S. number	Author, year	Study location	Study type	Technologies involved	Digital twin type	Application	Challenges	Justification for inclusion
1.	Cho et al., 2021 [5]	South Korea	Experimental study	• CBCT (Asahi Alphard 3030) • Facial scan (RAYface) • FaceGide program	Static anatomical craniofacial digital twin	• Measuring the sagittal position of maxillary central incisors relative to the forehead inclination • Fusion of CBCT and facial scan data for integrated soft‐hard tissue analysis for orthodontic diagnosis, evaluation, and treatment planning	• Small sample size which may have affected the statistical outcome, questioning the generalizability of the results	The merger of CBCT and facial scan of the patient allows a more detailed and individualized approach in orthodontic diagnosis, treatment planning, and assessing the facial attractiveness before and after the treatment, which can help in patient education and sustenance of motivation

2.	Pesapane et al., 2022 [11]	Italy	Narrative review	• AI • Wearables	i. Radiology digital twin (digital device)	• A virtual replica of radiology imaging devices such as CT, MRI, and X‐ray machines • Used for remote monitoring and predictive maintenance, ensuring machines function optimally and reducing downtime • AI‐powered fault detection systems analyze equipment performance and suggest repairs before failures occur	• Collection of data of both healthy and diseased subjects—in large quantities and consistently, from diverse populations is challenging • Lack of standardized training and validation of the technology yet	The digital twin of imaging devices improves the efficiency and longevity by predictive maintenance and remote monitoring by leveraging AI and real‐time data to reduce downtime and ensure continuous, high‐quality imaging services. A well‐monitored and regulated device can be a huge asset in orthodontics where obtaining a precise diagnosis is half the battle won
					ii. Patient‐specific clinical digital twin (digital patient)	• A virtual model of a patient’s anatomy, created using real‐time imaging data. (multiscale patient data) Helps in personalized diagnostics and treatment planning, improving accuracy in radiological assessments Can simulate disease progression, allowing radiologists to predict future health outcomes		Allows better precision in radiological assessments and also anticipate future health developments by studying the simulation of disease progression on imaging scans—can enable better understanding of how a skeletal derangement may progress if treated (or not treated) a certain way, which will be useful in orthodontic diagnosis, especially in cases with major skeletal discrepancy, going for either growth modulation or orthognathic surgery

3.	Lee et al., 2023 [7]	South Korea	Experimental study	• CBCT (Alphard 3030) • Desktop model scanner (Freedom UHD) • IOS: CS 3600 and i700 • R2GATE program for superimposition and distance measurement	Static anatomical digital twin (diagnostic simulation twin)	• Sagittal position analysis • Multimodal model fusion of CBCT and facial scan data for integrated soft‐hard tissue analysis for orthodontic diagnosis, evaluation, and treatment planning	• Manual placement of reference points may have introduced discrepancies between the control group and other groups • The exclusion of participants with prior orthodontic treatment, as appliances can cause artifacts and distort CBCT scans • IOS accuracy may be compromised by excessive light reflection from metal brackets and wires, along with saliva accumulation, which prolongs scan time	• Digital twins constructed from a combination of IOS + quickly scanned alginate impressions + CBCT allow an accurate digital dental representation, which holds immense potential in orthodontic and orthognathic procedures

4.	Attaran and Celik, 2023 [1]	USA	Narrative review	• IoT (the primary technology of every DT) • AI (includes robotics, image recognition, language recognition, neural networks, machine learning, and deep learning) • XR (includes immersive technologies like CR, AR, and MR) • CC	Three levels of integration of the digital twin as described by Kritzinger et al.: i. Digital model (simple digital representation) ii. Digital shadow (with a unidirectional information flow) iii. Digital twin (with bidirectional information flow)	Multi‐industrial applications described under manufacturing, aerospace/automotive, construction/real estate, utilities, agriculture, retail, mining, and the most upcoming—healthcare and dentistry Diagnosis and therapy • Preventive treatment • Drug development • Medical device utilization • Facility and operations design • Education and training	• High implementation costs—significant investment in hardware, software, and data infrastructure • Data security and privacy risks—real‐time connection between physical and digital assets increases vulnerability to cyberattacks • Integrating DTs with existing systems and platforms is technologically complex • Skill gaps and training needs in the technologies involved • DT systems require large volumes of high‐quality data to function accurately	• Provides detailed understanding of the integral technologies that form the core digital twin concept, which must be understood to be able to incorporate it in orthodontics as an important digital asset in the future. A better understanding of the underlying groundwork will allow better integration of it in orthodontics by simulation of similar procedures with different treatment goals—and further building up on that as the developmental model advances

5.	Cho et al., 2023 [4]	South Korea	Experimental study	• CBCT (Alphard 3030) • Digital facial scanners: RayFACE (RFS100), MegaGen SR305, and Artec Eva • R2GATE software • Geomagic Control X by 3D Systems	Static anatomical digital twin	• Monitoring growth and soft tissue changes, planning surgical treatment, and evaluating postop results • Fabrication of facial prostheses • Medical education and patient communication	• Scanner variability may affect the results • Each patient was scanned using only one scanner due to ethical concerns—prevented scanner comparisons on the same subjects • Lack of repeated measures so the precision of scanner could not be assessed • Limited number of standardized reference points	• Good‐quality soft tissue scans from the Artctec Eva enable realistic simulations, better patient communication, and personalized and outcome‐driven treatment planning. This technology supports accurate diagnosis, VTO, orthognathic planning, and objective posttreatment evaluation

6.	Moztarzadeh et al., 2023 [23]	Czech Republic	Experimental study	• MobileNetV2 • Blockchain • Metaverse Platform • IoMT • CC • PyTorch • Mobile DeepLabv3	CVM digital twin	• Automated diagnosis of the sixth stage of CVM from lateral cephalograms • Clinical decision support for orthodontic and maxillofacial treatments by identifying optimal treatment timing • Data traceability and integrity through blockchain • Virtual collaboration in metaverse among patients and clinicians	• Dataset size was only 319 images, which is relatively small and may limit generalizability • Dependence on ImageNet‐pretrained MobileNetV2 due to the lack of large medical datasets • Threshold sensitivity: accuracy affected by the 0.5 classification threshold; ROC analysis suggests improved performances with a higher threshold	• Evaluation of skeletal maturity status of the patient through evaluation of cervical vertebrae on the lateral cephalograms in an automated manner and with high accuracy. This is very important to know the patient’s skeletal maturity status and amount of growth remaining and thereby choosing the right orthodontic treatment plan accordingly

7.	Grieves, 2023 [21]	USA	Narrative review	• CAD • IoT • AR and VR • AI and ML	i. Digital twin prototype (DTP)	Represents potential product variations before physical production. Virtual simulation of products before manufacturing	• Even today, intelligent DTs demand massive computing resources, especially for real‐time simulations • High set‐up cost (sensors, platforms, and models) • The fallacy that DTs can only exist after a physical counterpart is created—this delays adoption in the design phase where DTs are most valuable • The Grieves Test of Virtuality (visual, performance, reflectivity, and prediction) is a benchmark test but hard to fully pass in all domains	• Provides detailed explanation of DTT development right from the lowest level to its transition from tangible products to biological systems. To enable large‐scale integration of this technology into intangible and abstract processes that orthodontics and dentofacial orthopedics naturally comprise of, the baseline is necessary to be understood—to replicate and even developmentally advance the DTs in our field, especially in today’s rapidly evolving era
					ii. Digital twin instance (DTI)	Captures real‐time data of a specific product in use. Real‐time monitoring of individual products		
					iii. Digital twin aggregate (DTA)	Collects and analyzes data from multiple DTIs for large‐scale insights		

8.	Ma et al., 2024 [12]	China	Narrative review	• CBCT • 3D cephalometric imaging • IOS • AI • MR, AR, and VR • Cloud computing platforms • Large language models	Patient‐specific digital twin	Primary focus of the application is orthodontics. Prediction of skeletal growth and personalized treatment optimization	• Lack of model transparency and interpretability • Data privacy and security concerns • Limited data standardization • High resource demand and computational complexity • Current lack of real‐world validation	The only review describing the possible future perspective of the DTT and its potential in orthodontics—helping in forecasting craniofacial development and improving orthodontic interventions
9.	De Domenico et al., 2025 [20]	Italy	Perspective article	• Mechanistic, generative, and multiscale models • AI and DL • Bioinformatics (multiomics, transcriptomics, social media, and knowledge graphs) • Network science (multilayer networks, adjacency tensors, and hypergraphs) • Comparative biological tools (organoids)	1. Specialized DT	• Focuses on specific subsystems or diseases • Example: artificial pancreas (Archimedes) program for diabetes management	• Unrealistic data requirements; vast, possibly unattainable, multiscale biological data • Computational infeasibility: complex, nonlinear models • Unique host‐microbiome and host‐pathogen interactions affect generalizability of predictions • Exposome is too complex to be accurately recorded, involving full lifetime environmental, dietary, and psychosocial exposures • Hidden variables like lifestyle changes may reduce personalization potential Limitations of ML models: They do not generalize well to untrained conditions • May suggest suboptimal treatments	• Provides theoretical and methodological foundation for applying DTT in orthodontics to evolve it into a personalized, predictive, and data‐driven care • Recognizing the importance of multiscale and mechanistic models enables the creation of orthodontic digital twins that realistically simulate how teeth, bone, and soft tissues respond to treatment forces. This approach connects generic AI frameworks with personalized orthodontic care, enhancing predictive accuracy, biological validity, and long‐term treatment success
					2. Nonspecialized DT	• Whole‐organism system‐level integration. These are designed to handle: Interconnected biological systems (for example, immune, cardiovascular, and neural systems) • Patient exposome (lifetime environmental and behavioral influences)		
					3. Mechanistic model‐integrated DT	• In silico frameworks that explicitly replicate causal biological mechanisms, incorporating: Gene regulatory networks • Protein–protein and metabolic interactions • Multiscale dynamics using Boolean or agent‐based or ODE models • Scenario‐based intervention simulations		

### 3.1. Study Characteristics and Quality Assessment

The studies included in this review were published between January 2005 and August 2025. Using a search strategy based on the PCC framework, nine studies were identified as relevant to the scope of this review, focusing on the applicability of DTT in orthodontics and dentofacial orthopedics. These studies explored the DTT and its direct and implied applicability in the field of orthodontics. All selected studies underwent quality appraisal using JBI tools. The overall quality was rated as moderate primarily due to the limited justification of study design, unclear sampling strategies, and insufficient discussion of confounding variables. These issues may limit the reproducibility and reliability of findings. Despite these limitations, the included studies generally supported the potential of DTT in revolutionizing orthodontics by enhancing diagnosis, treatment planning, and overall clinician and patient experience (Table 3).

**Table 3:** Critical appraisal criteria for eligible studies using the Joanna Briggs Institute (JBI) tool.

Experimental studies	Were the two groups similar and recruited from the same population?	Were the exposures measured similarly to assign people to both exposed and unexposed groups?	Was the exposure measured in a valid and reliable way?	Were confounding factors identified?	Were strategies to deal with confounding factors stated?	Were the groups/ participants free of the outcome at the start of the study (or at the moment of exposure)?	Were the outcomes measured in a valid and reliable way?	Was the follow‐up time reported and sufficient to be long enough for outcomes to occur?	Was follow‐up complete, and if not, were the reasons to loss to follow‐ up described and explored?	Were strategies to address incomplete follow‐up utilized?	Was appropriate statistical analysis used?
Cho et al. [5]	+	+	+	UC	−	+	+	−	−	−	+
Lee et al. [7]	+	+	+	−	−	+	+	UC	−	−	+
Cho et al. [4]	+	+	+	−	−	+	+	UC	−	‐−	+
Moztarzadeh et al. [23]	UC	UC	UC	−	−	UC	+	−	−	−	+

Review studies	Is the generator of the narrative a credible or appropriate source?		Is the relationship between the text and its context explained? (where, when, who with, and how)		Does the narrative present the events using a logical sequence so the reader or listener can understand how it unfolds?		Do you, as reader or listener of the narrative, arrive at similar conclusions to those drawn by the narrator?		Do the conclusions flow from the narrative account		Do you consider this account to be a narrative?
Grieves [21]	+		UC		UC		+		+		+
Ma et al. [12]	+		+		+		UC		−		+
De Domenico et al. [20]	+		+		+		+		−		+
Pesapane et al. [11]	+		+		−		+		+		−
Attaran and Celik [1]	+		UC		+		+		+		+

*Note*: Low risk (+), high risk (−), and unclear (UC).

### 3.2. Finding of Individual Studies

The studies reviewed highlight various applications of DTT in orthodontics, highlighting its emerging potential as a powerful tool for in‐depth diagnoses, personalized treatment planning, and predictive simulations.

Static anatomical DTs in orthodontics: Cho et al. [5] demonstrated the use of CBCT and facial scans to create a craniofacial DT to measure the sagittal positions. However, this study had a small and geographically specific sample size limiting its generalizability. Moreover, due to the lack of bidirectional information flow, such models fail to strictly fall under the category of DTs. Lee et al. [7] expanded on this by incorporating IOS data and a diagnostic simulation twin model, which allowed better fusion of 3D models for orthodontic analysis. They suggested that the use of hybrid models involving CBCT, IOS, and alginate impressions allows for greater accuracy. Similarly, Cho et al. [4] employed multiple digital facial scanners along with CBCT but found that scanner variability may have affected the results.

Patient‐specific DTs and clinical simulations: Attaran and Celik [1] discussed patient‐specific clinical DTs built using real‐time imaging data and AI, which can help simulate the progression of disease and improve diagnostic accuracy. They highlighted the advantages of these twins primarily in healthcare management, while Ma et al. [12] further focused on the applicability of DTs in orthodontics in an extensive manner.

Emerging frameworks and levels of DT maturity: Attaran and Celik [1] classified the developmental levels of DTs into three hierarchical levels: digital model, digital shadow (unidirectional data flow), and the DT (bidirectional data flow). Liu et al. [22] expanded on this by describing a maturity hierarchy of five levels: digital model, digital shadow, DT, cognitive twin, and federated twin. They emphasized that the higher‐level model enables better real‐time control, decision‐making, and system‐level integration—although current DT applications remain in their infancy and require further development.

**Figure 2:**
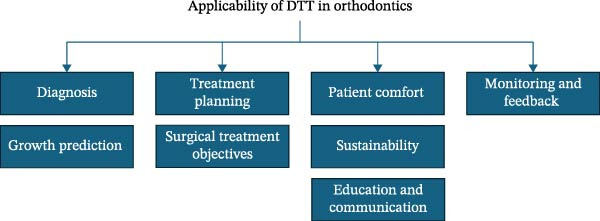
Applicability of digital twin technology in orthodontics.

**Figure 3:**
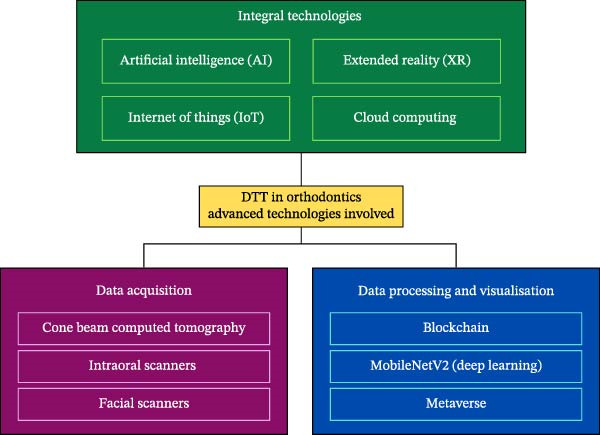
Advanced technologies involved in the applicability of digital twin technology in orthodontics: A functional relationship.

Integration of advanced technologies: Motarzadeh et al. [23] explored the application of DTs built on mobile neural networks, blockchain, and metaverse platforms to assess cervical vertebrae maturity (CVM) stages. Their AI‐based CVM DT achieved an accuracy of 82.13% and showed strong potential for further diagnostic capabilities. Ma et al. [12] highlighted the integral technologies used in DTT, primarily AI, IoT, and XR, which further include VR, AR, MR, and cloud computing (Figures 2 and 3).

## 4. Discussion

Michael Grieves was the first to unveil the conceptual underpinning of DTs in 2002 at a Society of Manufacturing Engineering (SME) conference in Troy, Michigan, where it was referred to as the “Conceptual Ideal for Product Lifecycle Management (PLM)” and lacked any formal name. Grieves further built on this concept in various academic and industrial settings, and the development led to the name “Digital Twin” being officially conceived in 2010 through collaboration with NASA. Notably, since their inception, the essential elements of the DT model—a physical object, its virtual equivalent, and the communication link that links them—have largely remained consistent [21]. The incorporation of patient‐specific digital reproductions can significantly improve diagnostic precision, treatment simulation, and individualized care planning, making Grieves’ early observations quite pertinent to contemporary applications even in healthcare and orthodontics. Although DTs have been widely used in manufacturing and, more recently, in healthcare, they are still not widely used in the field of dentistry and even less so in specializations such as orthodontics. The definitions of the DT concept have evolved through the years [25] in accordance with the level of development. An updated compilation is put together in Table 4. Among the various definitions proposed in the literature, Malik and Brem [51] conceptualization of a DT best applies to orthodontics as it captures the dynamic, evolving nature of tooth movement and skeletal and soft tissue changes alike during the treatment while emphasizing the ability to forecast future outcomes—which is central to orthodontic diagnosis, treatment planning, and monitoring.

**Table 4:** Existing definitions of digital twin technology.

S. number	Authors, year	Definition
1	Michael, 2015 [27]	“A set of virtual information constructs that fully describes a potential or actual physical manufactured product from the micro atomic level to the macro geometrical level. At its optimum, any information that could be obtained from inspecting a physical manufactured product can be obtained from its Digital Twin. The Digital Twin concept model contains three main parts: a) physical products in Real Space, b) virtual products in Virtual Space, and c) the connections of data and information that ties the virtual and real products together”
2	Ríos et al., 2020 [28]	“A product equivalent digital counterpart that exists along the product life cycle from conception and design to usage and servicing, knows the product past, current and possible future states, and facilitates the development of product related intelligent services”
3	Rosen et al., 2015 [29]	“A very realistic model of the current state of the process and their own behavior in interaction with their environment in the real world”
4	Schroeder et al., 2016 [30]	“A virtual representation of the real product. It has product’s information from the beginning of the life until the disposal of the product. The Digital Twin is a counter part of the physical device, machine or product in a CPS. It has the information related to the whole life cycle of a product”
5	Alam and El Saddik, 2017 [31]	“The cyber layer of CPS, which evolves independently and keeps close integration with the physical layer”
6	Brenner and Hummel, 2017 [32]	“A digital copy of a real factory, machine, worker, etc., that is created and can be independently expanded, automatically updated as well as being globally available in real time”
7	Ciavotta et al., 2019 [33]	“A digital avatar encompassing CPS data and intelligence, representing structure, semantics, and behaviour of the associated CPS, and providing services to mesh the virtual and physical worlds”
8	Gräßler and Poehler, 2018 [34]	“A cyber‐physical device of its own, which is connected to the CPPS and tries to emulate the human employee through dynamically adapted values of a database, which represent for example properties, preferences, work schedule and skillset”
9	Zhang et al., 2017 [35]	“A set of realistic product and production process models linking enormous amounts of data to fast simulation and allowing the early and efficient assessment of the consequences, performance, quality of the design decisions on products and production line”
10	Negri et al., 2017 [36]	“A virtual and computerized counterpart of a physical system that can exploit a real‐time synchronization of the sensed data coming from the field and is deeply linked with Industry 4.0”
11	Schleich et al., 2017 [37]	“A bi‐directional relation between a physical artefact and the set of its virtual models, enabling the efficient execution of product design, manufacturing, servicing, and various other activities throughout the product life cycle”
12	Schluse et al., 2017 [38]	“A one‐to‐one virtual replica of a “technical asset” (e.g., machine, component, and part of the environment). A digital twin contains models of its data (geometry, structure, …), its functionality (data processing, behaviour, …), and its communication interfaces. It integrates all knowledge resulting from modelling activities in engineering (digital model) and from working data captured during real‐world operation (digital shadow). A Digital Twin contains models of its “data” (geometry, structure, …), its functionality (data processing, behaviour, …) and its communication interfaces”
13	Söderberg et al., 2017 [39]	“A unique instance of the universal Digital Master model of an asset, its individual Digital Shadow and an intelligent linkage (algorithm, simulation model, correlation, etc.) of the two elements above”
14	Liu et al., 2023 [26]	“A digital representation that contains all the states and functions of a physical asset and has the possibility to collaborate with other digital twins to achieve a holistic intelligence that allows for decentralized self‐control”
15	Yun et al., 2017 [40]	“A perfect digital entity of a physical system; it accurately reflects the status of the corresponding physical machine. We can tightly control the system through a digital twin, that is a cyber model of the machine”
16	Autiosalo et al., 2021 [41]	“The cyber part of a Cyber‐Physical System”
17	Asimov et al., 2018 [42]	“A virtual replica of real physical installation, which can check the consistency for monitoring data, perform data mining to detect existing and forecast upcoming problems, and which uses an AI knowledge engine to support effective business decisions”
18	Bao et al., 2018 [43]	“A virtual model in the virtual space, and it is used to simulate the behaviour and characteristics of the corresponding physical object in real time”
19	Lee et al., 2022 [44]	“A near real‐time digital image of a physical object or process that helps optimize business performance. Two concepts of IoT (Internet of things) and IoS (Internet of Service) are combined to realise the smart factory based on a digital twin”
20	Haag and Anderl, 2018 [45]	“A comprehensive digital representation of an individual product. It includes the properties, condition, and behaviour of the real‐life object through models and data. The digital twin is a set of realistic models that can simulate its actual behaviour in the deployed environment. The digital twin is developed alongside its physical twin and remains its virtual counterpart across the entire product life cycle”
21	Luo et al., 2018 [46]	“A complete virtual prototype of an entire system and a one‐to‐one mapping relationship. Therefore, a multi‐domain digital modelling method is needed; a consistent model between the designed and the actual environment of a machine tool should be established, which needs the real‐time and accurate data mapping method; an effective machine learning algorithm to mine the data gathered from sensors and the control system is also necessary”
22	Nikolakis et al., 2018 [47]	“A digital replica of the physical environment along with the operator. This model constrains the behaviour of the twin towards replicating the actions of the physical system’s actuators”
23	Tao et al., 2019 [24]	“A set of virtual models. These mirror images and mapping of the physical products in the virtual space. They could reflect the whole life cycle process, as well as simulate, monitor, diagnose, predict, and control the state and behaviours of the corresponding physical entities. The virtual models include not only the geometric models, but also all rules and behaviours, such as material properties, mechanical analysis, health monitoring”
24	Hu et al., 2021 [48]	“A living model that continually adapts to change in the environment or operation using real‐time sensory data and can forecast the future of the corresponding physical assets for predictive maintenance”
25	Zhuang et al., 2018 [49]	“A dynamic model in the virtual world that is fully consistent with its corresponding physical entity in the real world and can simulate its physical counterpart’s characteristics, behaviour, life, and performance in a timely fashion”
26	Leng et al., 2019 [50]	“Each physical device will have its cyber part as a digital representation of the real device, culminating in the digital twin models. So, the digital twin can monitor and control the physical entity, while the physical entity can send data to update and synchronize its virtual model”
27	Liu et al., 2023 [26]	“A virtual representation of real‐world entities and processes, synchronized at a specified frequency and fidelity” which was introduced by in the Digital Twin Consortium in 2020”
28	Liu et al., 2023 [26]	A digital replica of real‐world entities and processes, synchronized at a specified frequency and fidelity”
29	Malik and Brem, 2020 [51]	A living model of the physical asset or system, which continually adapts to operational changes based on the collected online data and information and can forecast the future of the corresponding physical counterpart
30	Lo et al., 2021 [52]	A software representation of a physical asset, system, or process designed to detect, prevent, predict, and optimize through analytics to deliver business value
31	Korotkova et al., 2023 [53]	A virtual representation of a physical asset or system that securely holds all relevant static and dynamic information from concept to decommissioning, enabling high‐value collaborative services
32	Hariri‐Ardebili et al., 2023 [54]	A virtual replica of a physical asset, such as a dam, that can be used to simulate and test various scenarios, monitor performance, and optimize operations

### 4.1. DTs in Orthodontics

The studies included in this review collectively demonstrate specific applications of DTT in orthodontics, while others remain implicit and are still emerging within the field. With recent technological advancements, a blockchain‐based DT model was used in a study to evaluate CVM from lateral cephalometric radiographs using MobileNetV2 [23]. The authors drew attention to the expanding use of AI, IoT, and cloud computing in digital orthodontics in the review by Ma et al. [12]. Furthermore, Sabri [55] emphasized how standard diagnosis often tends to overlook dynamic smile esthetics. However, for today’s appearance‐conscious patients, DTs can bridge the gap between soft tissue esthetics and hard tissue emphasis by combining 3D facial scans, videos, and AI to objectively evaluate esthetics in both static and functional contexts [55]. The underlying idea of DTs is the logical development from static digital models to dynamic, interoperable ones [56, 57], thereby enabling improved understanding and predictive foresight for clinicians and patients. Lee et al. [7] demonstrated that combining CBCT with intraoral scans creates clinically useful DTs, offering a promising alternative to traditional diagnostic models. Other studies, such as those by Cho et al. [4] and Ma et al. [12], examined facial scanning technologies and AI‐based predictive models, underscoring the potential for personalized treatment optimization. There is a relative dearth of more elaborated studies focusing on orthodontics—so, looking ahead, several perspective trends appear to be building on this that are likely to shape the future integration of DTT in orthodontic practice. The present scoping review attempts to map and connect these emerging orthodontic applications of DTs with the broader body of existing and evolving evidence in dentistry to clarify current knowledge gaps and future research directions.

### 4.2. Drawing Parallels Between DTs in Other Sectors and Orthodontics

While our focus lies in the dental arena, DTT has integrated well into other industries, reiterating its importance in a multisector view. Industrial uses focus on cyberphysical systems that support stable production, sustainable agriculture, and urban transport. Manufacturing extensively leverages DTT by combining IoT sensors, computer‐aided designing (CAD) models, and simulation tools for real‐time monitoring, predictive maintenance, and lifecycle optimization [56–59]. Orthodontics could adopt a similar model, using patient‐specific simulations for tailored therapy, early error detection, and dynamic treatment planning with simultaneous model updates during treatment duration. Simulated testing in various scenarios, which is common in manufacturing, could analogously enable orthodontists to test alternative biomechanics or appliances virtually before applying them in patients [60, 61]. This multisector perspective illustrates the transformative potential of this technology, reinforcing its promising future in orthodontics [62].

### 4.3. Implementation Challenges and Future Scope

A prominent limitation of DTT in orthodontics is its major reliance on highly accurate and multimodal data. The accuracy of the entered data is an eminent prerequisite for correctness in the processes and the validity of the results obtained [6, 63, 64]. Similarly, imprecisions from imaging, scanning, or integration errors can lead to misleading simulations and thereby unreliable treatment predictions [12, 20]. Maddahi and Chen [63] emphasize that dentistry schools have not yet extensively adopted DTT for clinical or instructional objectives. Significant investments like devices, software subscriptions, updates, staff training, and more implementation costs add to the financial load [3, 62]. In addition, variability in data quality and lack of standardization across platforms further compromise the fidelity and clinical usefulness of DT models. The need for impermeable digital security and infrastructural requirements forms one of the main limitations for efficient implementation [57]. Furthermore, the lack of a consensus in definitions has led to confusion across disciplines regarding what truly constitutes DT in the most comprehensive manner, without ambiguity [65]. Some studies are not inherently orthodontic in focus but have been included due to their implied applicability and integrability within orthodontics. In the existing literature, often, the concept of DTs has been used interchangeably with simulation or digital dental modeling, despite the most defining feature being their live data coupling with physical assets. However, the road ahead seems hopeful with more advancements getting incorporated in the field of orthodontics—especially in the present clinical landscape with ever‐increasing awareness and rapid technological advancements. Key future research areas include developing semantic models for agent reasoning, aligning with standards like the semantic web and ISO 12967, and enhancing simulation for decision‐making, especially considering the time‐sensitive nature of most healthcare decisions [66]. Ethical concerns also need to be addressed. Enhanced data management with AI‐driven intelligence and patient‐specific algorithms, all across systems with robust security networks, particularly blockchain‐based systems, is expected in the future [5, 22]. A notable limitation of this review is the absence of a universally accepted definition of a DT in the orthodontic context. As a result, included studies represent a spectrum of DT maturity, ranging from fully dynamic bidirectional systems to models that approximate but do not strictly fulfill the criteria of being a DT. While this heterogeneity was addressed where identifiable within the review, it remains an inherent limitation of synthesizing evidence in a context where definitional consensus has yet to be established.

### 4.4. DTT in Orthodontics in the Context of AI‐Driven Scientific Synthesis

It is worth acknowledging that this scoping review was conducted at a time when the role of human‐authored reviews itself is under scrutiny. As discussed by Thurzo [67], the growing capabilities of AI‐driven synthesis tools have prompted meaningful debate about whether traditional reviews remain necessary or whether they are being rendered redundant by systems that can summarize literature at a larger and much faster scale. Yet, such tools currently fall short where evidence is heterogenous, terminology is contested, and domain‐specific judgement is required—conditions which precisely characterize the existing literature on DTT in orthodontics. The inconsistent application of the term “digital twin” across included studies, as discussed earlier in this review, is itself an example of the kind of conceptual problem that demands critical human appraisal rather than automated aggregation of information—at least in the current scenario, even though it is anticipated to change by 2030 [67, 68].

### 4.5. Clinical Significance

The DTT is clinically important as it enables the creation of patient‐specific virtual models that integrate real‐time anatomic, biomechanic, and imaging data. This ultimately allows precise and personalized diagnoses, treatment planning, and prediction of orthodontic treatment outcomes. These include prediction of tooth movement and associated prerequisites such as accurate and individualized bracket positioning or more controlled aligner planning. It supports dynamic monitoring and adjustment of treatment progress while increasing accuracy and reducing risks of errors. Furthermore, it facilitates better patient communication by visualizing expected results and enhances clinical workflows through data‐driven decision‐making [1]. Despite the present challenges, including standardization of data and high implementation costs, the ability of DTs to simulate complex orthodontic scenarios and adapt treatments in real time holds significant promise for improving treatment efficacy and overall patient experience [12].

## 5. Conclusion

The rapidly advancing digitization of a multitude of processes in orthodontic dentistry is encouraging novel yet stable steps into a new era of XR and real‐time monitoring like never before. The DTT is set to provide immense benefits and superior efficiency when it comes to orthodontic diagnosis and treatment planning. While it is still in its early stages, the established frameworks and challenges addressed in various industries, including healthcare, can provide a useful and clinically relevant roadmap. While the technology demonstrates nascent promise for orthodontic applications through a limited number of studies, including review papers, its clinical application remains in the preliminary stages. Nevertheless, more extensive adoption is anticipated in the future, contingent upon robust evidence from meticulously designed investigations, to further substantiate its capacity for augmenting patient care and clinical efficiency.

**Nomenclature**
DTT: Digital twin technology
DT: Digital twin
DTs: Digital twins
CBCT: Cone beam computed tomography
AI: Artificial intelligence
3D: Three‐dimensional
JBI: Joanna Briggs Institute
IOS: Intraoral scanner
CVM: Cervical vertebrae maturity
IOT: Internet of Things
XR: Extended reality
AR: Augmented reality
VR: Virtual reality
CAD: Computer‐aided designing
CAM: Computer‐aided manufacturing.

## Author Contributions

Presentation of the idea: Isha Bhardwaj. Study conceptualization: Isha Bhardwaj and Nidhin Philip Jose. Study design: Isha Bhardwaj, Nidhin Philip Jose, and Supriya Nambiar. Title and abstract screening: Isha Bhardwaj, Nidhin Philip Jose, and Dhruv Ahuja. Data extraction: Isha Bhardwaj, Dhruv Ahuja, and Shushma Rao. Writing: Isha Bhardwaj. Data analysis: Isha Bhardwaj, Dhruv Ahuja, and Shushma Rao. Reviewing the manuscript draft: Nidhin Philip Jose, Supriya Nambiar, and Sabarinath Prasad. Guidance and supervision: Nidhin Philip Jose, Supriya Nambiar, and Sabarinath Prasad.

## Funding

This study received no financial support from any funding agency.

## Disclosure

The OSF Registration DOI is https://doi.org/10.17605/OSF.IO/3QFKJ.

## Ethics Statement

The authors have nothing to report.

## Consent

The authors have nothing to report.

## Conflicts of Interest

The authors declare no conflicts of interest.

## Supporting Information

Additional supporting information can be found online in the Supporting Information section.

## Supporting information


**Supporting Information** Supporting Information mentioned in the manuscript is uploaded separately. Table S1: PRISMA ScR Checklist for scoping reviews. Table S2: Search strategy.

## Data Availability

The datasets used and/or analyzed during the current study are available from the corresponding author upon reasonable request.
